# Molecular basis of halorespiration control by CprK, a CRP-FNR type transcriptional regulator

**DOI:** 10.1111/j.1365-2958.2008.06399.x

**Published:** 2008-08-20

**Authors:** Colin Levy, Katharine Pike, Derren J Heyes, M Gordon Joyce, Krisztina Gabor, Hauke Smidt, John van der Oost, David Leys

**Affiliations:** 1 Manchester Interdisciplinary Biocentre, The University of ManchesterManchester M1 7DN, UK; 2 Department of Biochemistry, University of LeicesterLeicester LE1 7RH, UK; 3 Laboratory of Microbiology, Wageningen University6703 HB Wageningen, The Netherlands

## Abstract

Certain bacteria are able to conserve energy via the reductive dehalogenation of halo-organic compounds in a respiration-type metabolism. The transcriptional regulator CprK from *Desulfitobacterium* spp. induces expression of halorespiratory genes upon binding of *o*-chlorophenol ligands and is reversibly inactivated by oxygen through disulphide bond formation. We report crystal structures of *D. hafniense* CprK in the ligand-free (both oxidation states), ligand-bound (reduced) and DNA-bound states, making it the first member of the widespread CRP-FNR superfamily for which a complete structural description of both redox-dependent and allosteric molecular rearrangements is available. In conjunction with kinetic and thermodynamic ligand binding studies, we provide a model for the allosteric mechanisms underpinning transcriptional control. Amino acids that play a key role in this mechanism are not conserved in functionally distinct CRP-FNR members. This suggests that, despite significant structural homology, distinct allosteric mechanisms are used, enabling this protein family to control a very wide range of processes.

## Introduction

Halogen containing compounds such as chlorinated ethenes and chlorophenols are among the most abundant pollutants in the soil, sediments and groundwater, mainly caused by past and present industrial and agricultural activities (Stringer and Johnston, 2001). Over a decade ago, solid evidence emerged that certain bacteria are able to conserve energy via the reductive dehalogenation of halo-organic compounds in a respiration-type metabolism (reviewed in Smidt and de Vos, 2004). Halorespiring bacteria can use a wide range of chloroalkenes (e.g. tetrachloro-ethene and trichloro-ethene) or chloroaromatic compounds (e.g. chlorophenols, chlorobenzenes and even dioxins) as the terminal electron acceptor. In view of their favourable degrading capacities, these halorespiring microorganisms most likely play a key role in the efficient biological remediation of halogenated hydrocarbons in anaerobic environments.

*Desulfitobacterium dehalogenans* and the closely related *D. hafniense* have been used as model organisms in halorespiration studies. They are strictly anaerobic Gram-positive bacteria that have the capacity to degrade ortho-chlorophenol, and represent a group of microorganisms that is frequently isolated from environmental samples. Genome sequences are available for *D. hafniense* strains Y51 and DCB-2 (Villemur *et al*., 2002; Nonaka *et al*., 2006), and for *Dehalococcoides ethenogenes* strain 195 and *Dehalococcoides* sp. CBDB1 (Kube *et al*., 2005; Seshadri and *et al.*, 2005). Sequence comparison reveals that a large number of the halorespiration-associated genes are present in multiple copies, presumably as a result of gene duplications and/or horizontal gene transfer events. The presence of these paralogues often correlates with an expanded substrate range (Smidt and de Vos, 2004; Villemur *et al*., 2006). Halorespiration-associated genes cluster in operon-like structures that contain, among others, the structural genes coding for the heterodimeric reductive dehalogenase, the key enzyme in the halorespiratory pathway responsible for reduction of the halogenated terminal electron acceptor. This enzyme belongs to one of the three currently recognized classes of B_12_-enzymes (Banerjee and Ragdale, 2003). In comparison to the other two B_12_-enzyme classes, little is known about the reductive dehalogenase enzyme class.

Intriguingly, the operon structure and content reveals significant variation occurs in both nature and quantity of associated genes encoding for protein maturation factors or transcriptional regulators. While the vast majority of reductive dehalogenase genes in *Dehalococcoides* spp. are associated with genes encoding either a MarR-type transcriptional regulator or a putative two-component signal transduction system (Kube *et al*., 2005; Seshadri and *et al.*, 2005), *Desulfitobacterium* spp. preferentially use a CRP-FNR type regulator, termed CprK to control halorespiration (Smidt *et al*., 2000). The CprK associated with the *o*-chlorophenolacetic acid (OCPA) reductive dehalogenase (CprAB) from *D. dehalogenans* and the closely related *D. hafniense* has been shown to bind OCPA with μM affinity leading to sequence-specific DNA-binding, and ultimately transcriptional activation of the associated *cpr* gene cluster (Pop *et al*., 2004; 2006; Gabor *et al*., 2006; Joyce *et al*., 2006; Mazon *et al*., 2007). Inactivation of CprK under aerobic conditions through Cys-11–Cys-200 disulphide bond formation is readily observed *in vitro*, although it remains unclear whether this process has physiological relevance (Gabor *et al*., 2006; Pop *et al*., 2006).

The CRP-FNR family of prokaryotic transcriptional regulators is very well studied, with the paradigm *E. coli* cAMP receptor protein (CRP or CAP) having attained text book status (Harman, 2001; Lawson *et al*., 2004). Members of this family are homodimeric and contain an N-terminal sensor domain linked via a long α-helix (involved in formation of the dimer interface) to the C-terminal helix–turn–helix (HTH) DNA-binding domain. These regulators have been shown to be involved in control of a wide range of processes, including oxidative stress response, stationary phase survival, anaerobic metabolism (fermentation, denitrification, nitrogen fixation, halorespiration), arginine catabolism, pathogenesis, CO oxidization, quorum sensing, flagellum biosynthesis and aromatics degradation (Green *et al*., 2001; Korner *et al*., 2003). However, despite the availability of crystal structures for individual family members [CooA (Lanzilotta *et al*., 2000), CRP (Passner *et al*., 2000), PrfA (Eiting *et al*., 2005)] as well as the CRP:cAMP:DNA and CRP:cAMP:DNA:αCTD complex structures (Benoff *et al*., 2002; Lawson *et al*., 2004), the allosteric mechanism by which ligand binding to the N-terminal domains induces conformational transition to the active, DNA-binding state through structural rearrangement of the C-terminal domains remains poorly understood. This is due to the lack of the corresponding ligand-free and ligand-bound structures for a given member of this superfamily.

Recent structure determination of the oxidized (Cys-11–Cys-200 cross-linked) *Desulfitobacterium hafniense* CprK-OCPA and a reduced, ligand-free *D. dehalogenans* CprK structure provided a first glimpse of the CprK allosteric mechanism. However, because the oxidized protein is inactive, and as the apo-structure is low-resolution, no detailed analysis beyond the OCPA binding site could be performed (Joyce *et al*., 2006). In the present study, five new *Desulfitobacterium hafniense* CprK structures are presented. Integration with biochemical/biophysical analyses of CprK has led to a detailed model for the allosteric rearrangements that cause the switch from an inactive (free) to and active (DNA-bound) state.

## Results and discussion

**Fig. 1:**
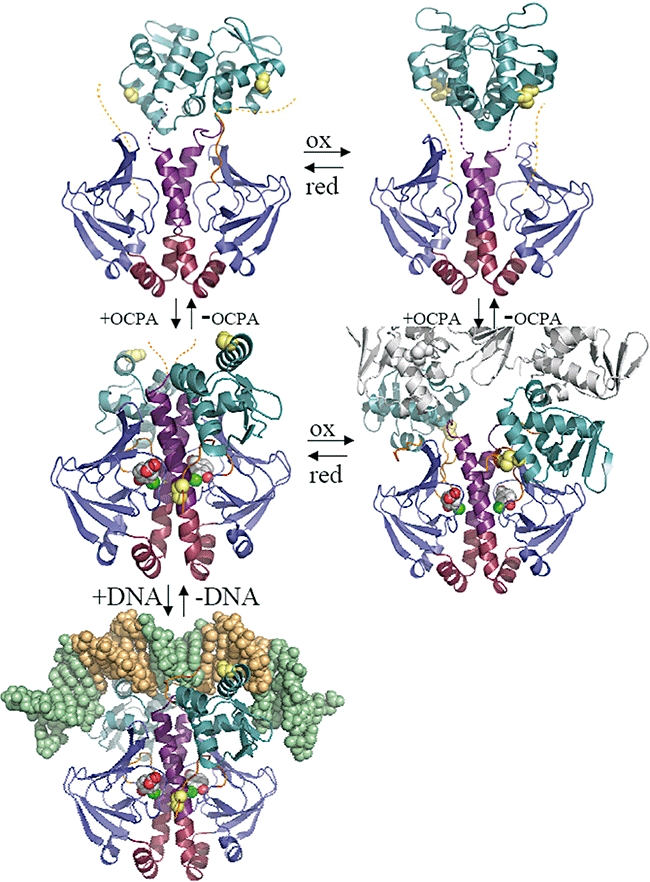
Atomic structures of *D. hafniense* CprK in different states. A representative dimer is depicted for each structure in cartoon form. Individual structural elements are coloured as follows, the N-terminus (residues 1–18) in orange, the effector β-barrel domain (19–107) in blue, the central α-helices not affected in position by ligand binding (108–127) in red, the central α-helix region affected by ligand binding + loop region in purple (128–148), the DNA-binding domain (149–227) in teal, the C-terminus (228–232) in orange. In addition, the position of C11 and C200 is indicated (when visible) by yellow spheres. The bound OCPA ligand is depicted in atom coloured spheres with grey carbons. The bound DNA is depicted in atom spheres with nucleotides constituting the (de)halobox sequence in pale orange. In case of the oxidized CprK:OCPA complex a symmetry-related molecule is depicted in grey to illustrate the changed quaternary structure in this particular state. Dotted lines indicate the position of highly mobile linker elements or N/C termini not visible in the electron density maps. Structures are organized as follows, top left: CprK_C200S_; top right: oxidized CprK; middle left: CprK_C200S_:OCPA; middle right: oxidized CprK:OCPA (previously determined 2H6B; Joyce *et al*., 2006); bottom left: (de)halobox DNA: CprK_C200S_:OCPA complex.

We here present crystal structures of the oxidized ligand-free (3.2 Å) and reduced ligand-free (2.0 Å), OCPA-bound (1.8 Å) and DNA-bound (1.8 Å) *D. hafniense* CprK in addition to an intermediate OCPA-bound structure obtained by soaking ligand-free crystals (2.0 Å) (Fig. 1). These structures are described in more detail below, and serve as the basis for gaining a more detailed insight into the allosteric behaviour of this transcriptional regulator.

### Crystal structure of OCPA-bound CprK_C200S_

**Fig. 2:**
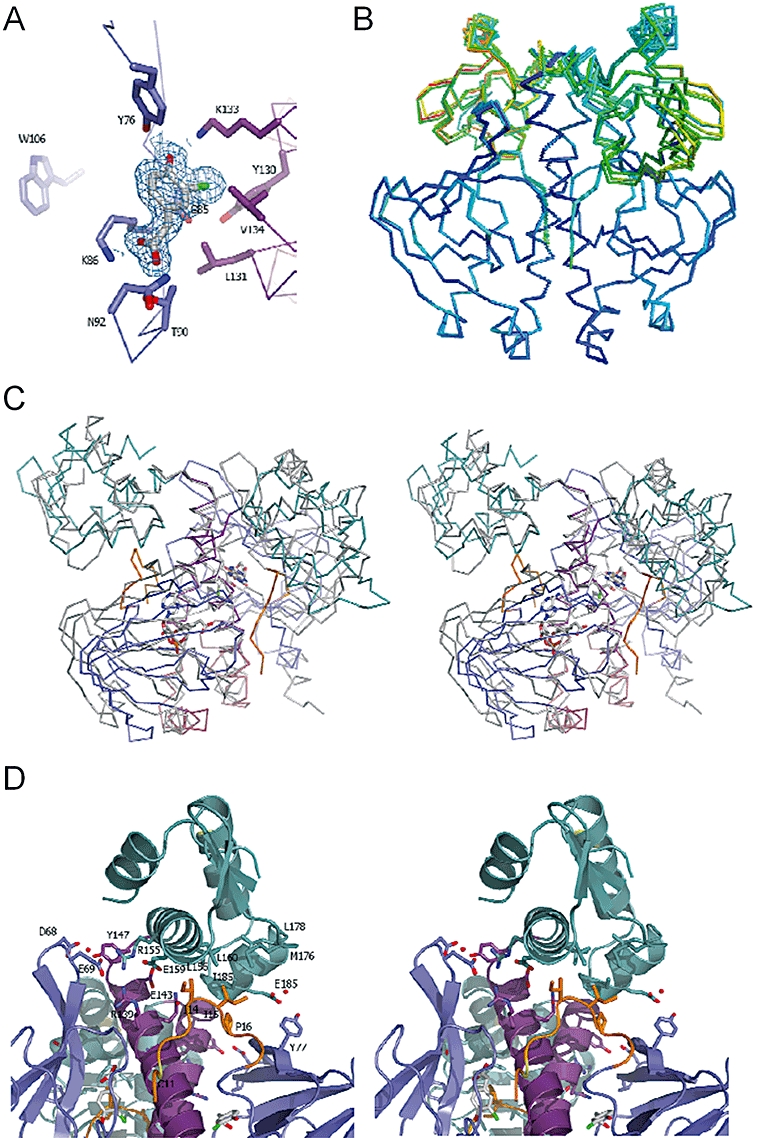
Structure of the CprK_C200S_:OCPA complex. A. OCPA binding site. Key residues involved in OCPA binding are shown in atom coloured sticks (coloured coded with carbons coloured according to Fig. 1). The σA 2F_o_F_c_ electron density map surround the bound OCPA molecule and is contoured at 1σ. In addition the Trp-106 side-chain is shown that is approximately ∼18 Å away from the bound OCPA. B. Overlay of distinct CprK_C200S_:OCPA dimer structures in two crystal forms (with and without glycerol). The ribbon traces are coloured according to Cα B-factor values. C. Overlay of CprK_C200S_:OCPA (in ribbon trace coloured according to Fig. 1) with the CRP:cAMP structure (in grey ribbon trace; PDB code 1G6N). The overlay is based on structural alignment of the respective central α-helices. D. Stereoview of the inter-domain contact established between a single DNA domain and the sensor binding domains. Key residues are depicted in atom coloured sticks, coloured coded as in Fig. 1.

In order to avoid formation of the inactive, oxidized CprK under aerobic conditions, the CprK_C200S_ mutant was used to obtain structural insights into the reduced CprK structure. Previous studies indicated that this mutation does not significantly affect CprK function and only has minor impact on DNA-binding affinity (Gabor *et al*., 2006; Joyce *et al*., 2006; Mazon *et al*., 2007). The 1.8 Å crystal structure of the CprK:OCPA_C200S_ complex contains two CprK dimers in the asymmetric unit, each dimer binding two OCPA ligands to the N-terminal sensor domains (Fig. 1). Similar to the previously determined oxidized CprK:OCPA complex, the phenol and acetic acid groups of OCPA interact with amino acids from both CprK monomers through extended hydrogen bonding networks. This positions the chloride atom within a hydrophobic pocket at the dimer interface (Joyce *et al*., 2006; Fig. 2A).

CprK_C200S_:OCPA crystals were analysed both in the presence and in the absence of glycerol cryoprotectant. This resulted in minor differences in crystal packing. Comparison of the individual monomers within the asymmetric unit of both crystal forms does not reveal significant structural differences for the entire β-barrel and associated α-helices of the N-terminal sensor domain (residues Asp-17 to Val-141) (Fig. 2B). Moreover, a comparison of the available individual CprK_C200S_:OCPA monomers reveals an identical conformation for the N-terminus, with the exact position of the different DNA-binding C-terminal domains only influenced to a minor degree by crystal packing (see Fig. 2B). Likewise, comparison with the previously published oxidized CprK:OCPA complex (PDB code 2H6B, to 2.2 Å) only shows minor structural changes of this region from Asp-17 to Val-141. It is concluded that the nature and the effects of ligand binding on this domain are independent of crystal packing. In contrast, the same comparison reveals the position of both the N-terminus (Met-1 to Pro-16) and the C-terminal DNA binding domains to be highly dependent on the CprK oxidation state. In contrast to the marked asymmetry of the oxidized CprK:OCPA structure, near-perfect twofold symmetry relates the monomer structures within a single CprK_C200S_:OCPA dimer. This arrangement places both HTH domains in a position compatible with binding to the palindromic (de)halobox DNA.

**Fig. 3:**
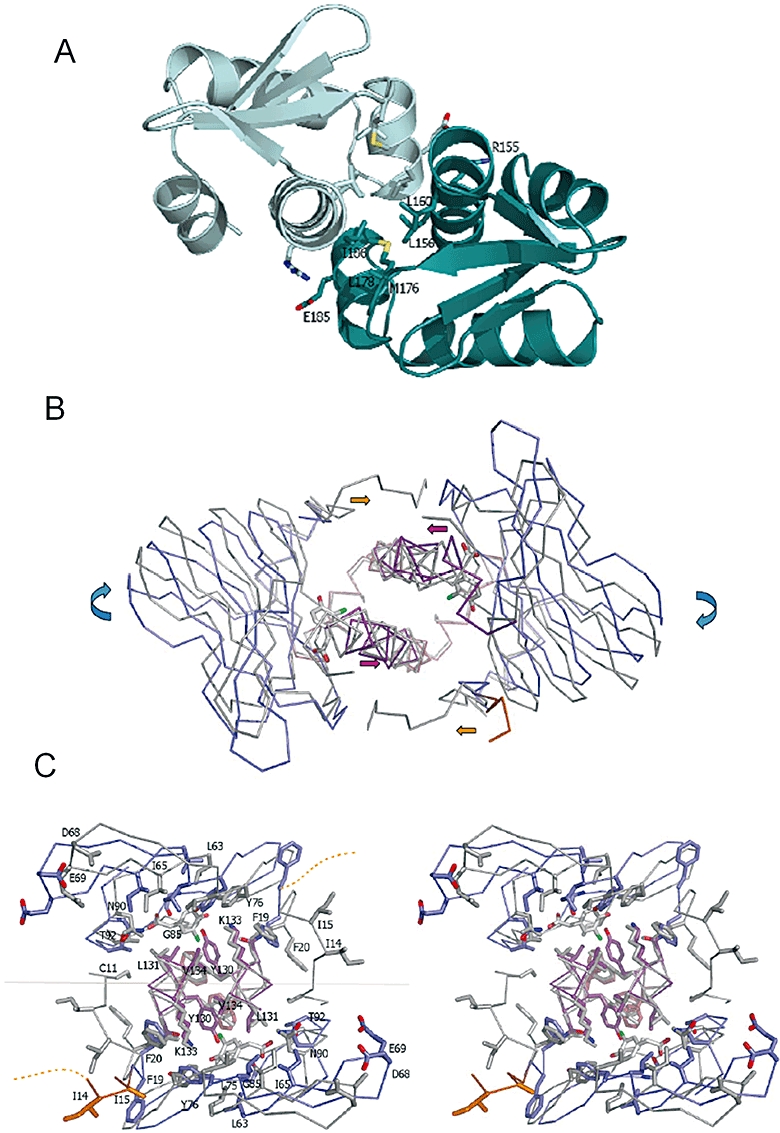
Structure of the ligand-free CprK_C200S_. A. DNA domain interface. A stereo view of the DNA-binding domain interface with key residues represented in atom coloured sticks. B. Overlay of the CprK_C200S_ ligand-free sensor domains (ribbon model coloured as in Fig. 1) with the corresponding CprK_C200S_:OCPA structure (in grey ribbons). Coloured arrows indicate relative motion of individual structural elements upon ligand binding. C. Stereoview of an overlay of the ligand binding sites and immediate environment for both CprK_C200S_ and CprK_C200S_:OCPA structures. For clarity, labels in the left panel indicate the position of CprK_C200S_ residues below the grey horizontal line and for CprK_C200S_:OCPA residues when above that line.

Analysis of the contacts made between the sensor domains and the DNA-binding domains might pinpoint residues that are involved in the allosteric effects of ligand binding on DNA affinity. The CprK_C200S_:OCPA dimer structure reveals that each individual CprK DNA-binding domain establishes a small contact area [approximately 270 Å^2^ with a surface complementarity of 0.691 (Lawrence and Colman, 1993)] with both N-terminal domains of the dimer. A large proportion of this contact area is centred around an extensive salt-bridge network established between the DNA-binding domain Arg-155 and Glu-159 with the central α-helix Arg-139 and Glu-143 residues in addition to Glu-68 (in the sensor domain) (Fig. 2D). In this case, Glu-68 is provided by one monomer while the other residues involved are derived from the opposite monomer. In addition, further inter-monomer polar contacts are formed between Tyr-147 (DNA-binding domain) and Glu-68 and Asp-69 (sensor domain). Within a single monomer, polar contacts between the sensor domain and the DNA-binding domain are limited to hydrogen bonds between Tyr-77 and Glu-185, and between Gln-140 and the Ile-186 backbone. In contrast, a significant set of hydrophobic interactions is established within each single monomer between residues Ile-14–Ile-15–Pro-16 from the N-terminus and the DNA-binding domain that leads to near-complete burial of both Ile side-chains. Surprisingly, the DNA-binding domain residues involved in this interaction (Leu-156, Leu-160, Leu-178, Met-176 and Ile-186) also establish the dimer interface formed between DNA-binding domains in the non-DNA binding forms of the protein (Fig. 3A). Furthermore, residues Arg-155 and Glu-185 (implicated in the CprK_C200S_:OCPA inter-domain salt-bridge network) form part of this DNA-binding domain dimer interface in the non-DNA binding form. Hence, a majority of the residues involved in establishing CprK inter-domain contacts in the OCPA bound form perform a similar role in the ligand-free CprK structure, albeit within a completely different contact network. Ligand-dependent changes in the position or conformation of those residues involved in only one network (e.g. Glu-68 and Asp-69) are thus likely to drive the conformational transition following ligand binding.

Surprisingly, comparison of the CprK_C200S_:OCPA complex with the evolutionary related CRP:cAMP complex (PDB 1G6N) reveals that the relative positions and hence inter-domain contacts are distinct (see Fig. 2C). While in CRP the vast majority of contacts between the N- and C-terminal domains are between domains from the same monomer (intramolecular; Passner *et al*., 2000), a significant proportion of the CprK contacts are between domains from different monomers (intermolecular). In addition, the hydrophobic contacts formed between the CprK N-terminal peptide (Asp-9–Pro-16; not present in CRP) and the corresponding DNA-binding domain of the same monomer have no counterpart in CRP.

### Crystal structure of the ligand-free CprK_C200S_

**Fig. 4:**
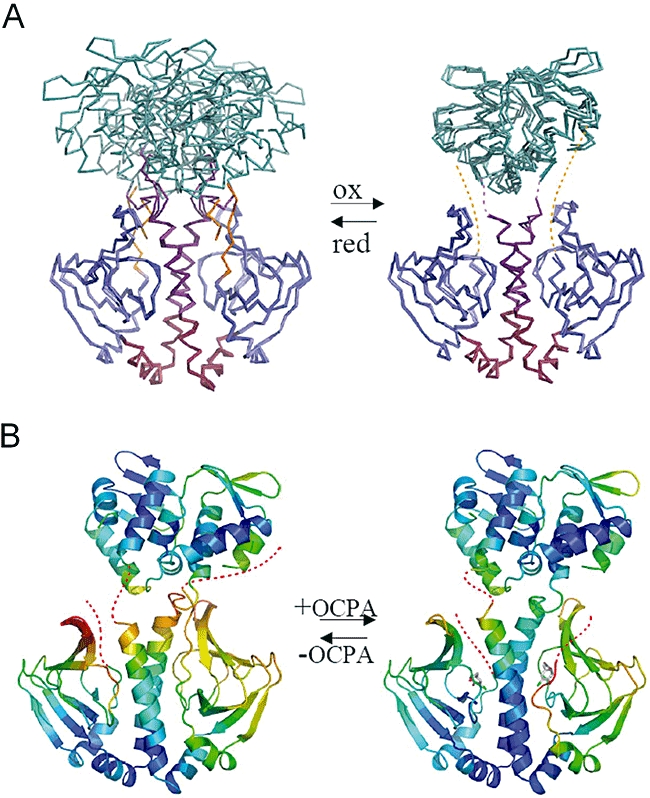
Dynamic information obtained from CprK crystal structures. A. Overlay of the different CprK ligand-free structures. The left panel displays an overlay of the *D. hafniense* CprK_C200S_ and *D. dehalogenans* CprK (PDB code 2H6C) depicted in ribbons coloured coded according for Fig. 1. The right panel displays a similar overlay but for the oxidized *D. hafniense* CprK structure. B. Comparison between the CprK_C200S_ ligand-free (to the left) and OCPA-soaked CprK_C200S_ (to the right) structures. The ribbon models are coloured according to Cα B-factors.

Crystals of the ligand-free CprK_C200S_ from *D. hafniense* diffract to significantly higher resolution (2.0 Å) and exhibit significantly less disorder than the previously determined ligand-free CprK structure from *D. dehalogenans* (2.9 Å; PDB code 2H6C; Joyce *et al*., 2006), allowing for more detailed comparison. In the *D. hafniense* crystal structure, a single CprK dimer is present in the asymmetric unit. Little to no contacts between the sensor domains and the DNA-binding domains can be observed (Fig. 1). Similar to previously determined crystal structures of non-DNA binding CprK forms (Joyce *et al*., 2006), the C-terminal domains contribute to the CprK dimer interface predominantly through hydrophobic interactions made between the D and E α-helices of both DNA-binding domains (Fig. 3A). Although DNA domain:DNA domain interactions have not yet been observed in other CRP-FNR family members, the fact that it is observed for all crystal structures of non-DNA binding CprK forms strengthens the notion that it is physiologically relevant for this regulator. In the *D. hafniense* crystal structure, the relative position of the DNA domains with respect to the sensor domains is drastically different when compared with the previous *D. dehalogenans* structure. Given the lack of significant interaction with the N-terminal domains and the observed disorder in the linker regions, the different positions observed for the DNA-binding domains are most likely a consequence of distinct crystal packing. This reflects the extreme mobility of these domains in the ligand-free, reduced state (Fig. 4A). The linker regions (Ala-142–Asn-148) between both CprK_C200S_ domains are highly mobile and no continuous electron density could be observed for the linker region of monomer A. Compared with the ligand bound CprK_C200S_ structures, the overall conformation of the individual sensor domains of the ligand-free CprK_C200S_ dimer displays slight differences (RMSD of 0.413 Å for all Cα as opposed to values 0.150 Å for the ligand bound structures). This reflects the increased impact of the different crystal packing contacts on the sensor domains that are relatively more flexible and less restrained than the ligand-bound forms. The ligand binding pocket is predominantly empty with several residues occupying either multiple or different conformations in both monomers. The N-terminal region is only visible from Phe-19 onwards for monomer A, while the Ala-13–Asn-18 polypeptide of monomer B is stretched towards the monomer A DNA domain. It is unlikely that the latter conformation accurately reflects the solution state as it appears significantly influenced by crystal packing. Nevertheless, it does illustrate how the inter-domain Cys-11–Cys-200 disulphide bridge can rapidly be formed under aerobic conditions due to the transiently occurring close proximity of both cysteines in solution.

### Comparison between ligand-free and ligand-bound crystal structures

Comparison between both ligand-free and ligand-bound CprK_C200S_ structures reveals that ligand binding affects the relative position of most residues (Fig. 3B and C). Nevertheless, a superposition of both structures reveals that the Ser-108–Phe-127 segment, which is a section of the central α-helix and the entire preceding α-helix, is not significantly perturbed upon ligand binding (RMSD 0.182 Å for Ser-108–Phe-127 Cα's as opposed to 1.017 Å for all Cα's from the sensor binding domain). In agreement with this observation, this segment also has a significantly lower average B-factor. Upon ligand binding, the position of both sensor domain β-barrels shifts with respect to the Ser-108–Phe-127 segment. The motion relating the distinct β-barrel position in both crystal structures constitutes a rigid body hinge movement around residue Ser-108 (Fig. 3B). Due to the position of the hinge point being close to the central α-helices, many of the direct contacts between these and the β-barrels remain intact. The largest change within the sensor domain concerns the loop connecting β-strands 4 and 5 (containing residues Glu-68 and Asp-69) that moves by approximately 4 Å. While the previous structural rearrangements can be described as a rigid body motion, a localized reorganization of the peptide backbone occurs within the ligand binding site upon OCPA binding. A large shift of 3.8 Å in the Cα position of Gly-85 directly results from accommodating the bulk of the ligand and establishing direct H-bonding to the phenolate group. In turn, this significant shift in position and conformation causes new hydrogen bonding interactions and direct contacts between Gly-85 and Tyr-130 and Leu-131. The position of the latter two residues is altered as a consequence, reducing the volume of the hydrophobic pocket to match the volume of the ligand chloride atom. At first glance, this reorganization appears unrelated to the rigid body β-barrel rearrangement as it could equally well have occurred within the confines of the ligand-free crystal structure. However, an artificial model of the CprK:OCPA structure in the absence of the ligand induced β-barrel hinge motion reveals that the ligand phenol hydroxyl group would be in a position too distant from the conserved Lys-133 to make a direct interaction. It therefore seems likely that, after the initial induced-fit reorganization of the binding pocket, formation of a phenolate–Lys-133 interaction and the correct positioning of the chloride atom within the hydrophobic pocket drive the rigid body motion.

The relative orientation as well as the conformation of the central α-helices is also subjected to ligand-induced changes, albeit less dramatic (e.g. a maximal shift of ∼0.9 Å in the position of Cα of Ala-142). As a consequence, the C-terminal regions of both central α-helices slide towards the dimer interface. This allows for the close approach of residues Ile-65 and Phe-67 (β4-β5 loop) with Ala-142 from the opposite monomer, a prerequisite for the sensor domain rigid body motion described (Fig. 3B). Moreover, the ligand-induced hinge motion leads to significant reorientation of the N-terminal polypeptide, with the side-chain of Phe-20 effectively occupying the position previously occupied by the Phe-19 side-chain (Fig. 3C). This releases the latter to form part of a small hydrophobic pocket at the dimer interface and close to the ligand binding sites where the N-terminal Cys-11 side-chain becomes buried. Thus, in concert with the rigid body reorientation of the sensor domains that affects the position of Glu-68 and Asp-69, the latter ligand-induced motion leads to ordering of the N-terminus and thus positions residues Ile-14–Ile-15–Pro-16. Both sets of residues are essential to the inter-domain contacts observed in the CprK_C200S_-OCPA structure. Ligand binding thus drives formation of a new network of inter-domain contacts between the DNA-binding domains and the N-terminal region, ultimately leading to enhanced DNA affinity.

### Crystal structure of OCPA-soaked CprK_C200S_

Comparison of crystal structures of distinct states of a protein to inform on the likely path of conformational changes occurring during inter-conversion between these forms cannot reveal whether the individual changes identified occur simultaneously, sequentially or randomly. However, a crystal structure of an intermediate species along the path can provide additional information that allows a more informed model for the inter-conversion. Crystals of the ligand-free CprK_C200S_ can be soaked with high concentrations of *o*-halogenated phenol acetic acid ligands without any apparent detrimental effects. The corresponding structures reveal that OCPA is bound in both sensor domains and establishes a near identical network of polar and hydrophobic interactions within the active site as described for the cocrystallized structures (Fig. 4B). A significant structural rearrangement of both sensor domains similar to that observed when comparing ligand-free and OCPA-bound CprK_C200S_ structures has occurred, but in absence of significant DNA-binding domain reorientation. While the conformation of the central α-helices (Ser-108 to Ala-142) has become identical to the OCPA cocrystallized structures, the sensor domain β-barrels have only partly rotated along the Ser-108 hinge points. This is likely due to the fact that full rotation as is observed for the OCPA-bound structures would lead to minor clashes with crystal symmetry related neighbouring molecules for both monomers. This suggests the reorientation of the α-helices can occur independent of the β-barrel motion (or alternatively, the individual changes observed here are not very energetically intertwined) and possibly is a prerequisite for the latter. While the N-terminal residue Phe-20 has effectively replaced the previous position of Phe-19 in both monomers, as occurs during conversion from the ligand-free to the ligand cocrystallized structure, the preceding N-terminal residues remain unstructured in both monomers. The complete reorganization of the N-terminal polypeptide should be possible as no clashes with crystal symmetry related neighbours can be observed for the corresponding, putative conformation. We therefore speculate that reorganization of the N-terminus only occurs during or following DNA-binding domain reorientation. Furthermore, in view of the similar resolution and near identical crystal packing, a direct B-factor comparison is feasible between the ligand-free CprK_C200S_ and corresponding OCPA-soaked structure. This reveals that ligand binding leads to significantly decreased B-factors for both β-barrels as well as the C-terminal section of the central α-helices (Fig. 4B, average decrease of ∼6 Å^2^). This analysis is in agreement with the recent mass spectrometry studies of the effects of OCPA binding on CprK (Mazon *et al*., 2007).

### Crystal structure of the oxidized ligand-free CprK

Crystals of the oxidized, ligand-free wild-type CprK were difficult to obtain and only yielded data to low resolution (to 3.2 Å) using microfocus beamlines (Fig. 1). Nevertheless, the relative position of the individual domains could be unambiguously determined and is roughly identical for the three individual dimers present in the asymmetric unit. As observed for the other ligand-free CprK crystal structures, both DNA-binding domains appear to act as a single unit by virtue of hydrophobic interactions between helices D and E. In contrast, whereas the position of the DNA-binding domain dimer appears largely unrelated to the position of the remainder of the protein in the reduced ligand-free CprK structures, the symmetry axis relating both N-terminal domains more or less coincides with that relating both DNA-binding domains in case of the individual oxidized ligand-free CprK dimers (Fig. 4A). This is most likely a consequence of the tight restraints on the respective positions by virtue of the intermonomer Cys-11–Cys-200 disulphide bridges. It thus appears that considerable flexibility in the DNA-binding domain positions, and presumably in the entire molecule as a consequence, is lost upon oxidization, confirming the previously observed effect of limited proteolysis as detected by mass spectrometry (Mazon *et al*., 2007).

### Kinetic and thermodynamic solution studies of OCPA binding by CprK_C200S_

**Fig. 5:**
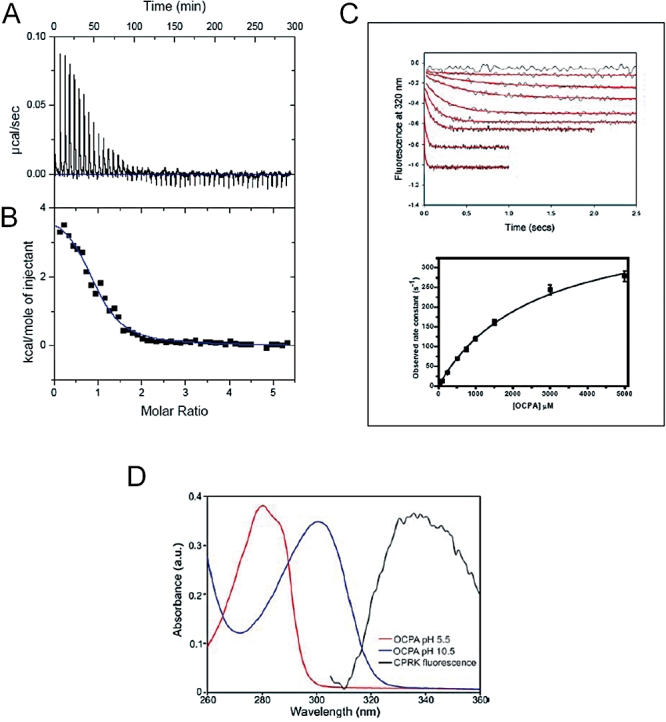
Solution data for OCPA binding by *D. hafniense* CprK_C200S_. A. ITC analysis of CprK_C200S_ OCPA interaction. The ITC experiment was carried out by titrating OCPA into the chamber containing CprK. This panel shows the raw heating power over time. B. The fit of the integrated energy values. C. Stopped-flow Trp fluorescence quenching. The top panel represents stopped-flow kinetic transients observed upon mixing various concentrations of OCPA with 1 μM CprK. An excitation wavelength of 295 nm was used and the fluorescence emission was measured using a 320 nm cut-off filter. Transients were recorded between 1 and 10 s at 0, 1, 5, 10, 25, 50, 100, 250 and 500 μM OCPA. The bottom panel depicts the OCPA concentration dependence of the observed rate. D. OCPA absorbance. Absorbance spectra of 5 μM OCPA were recorded in 25 mM Tris, 25 mM ethanolamine, 50 mM MES, 50 mM NaCl at pH 5.5 and pH 10.5. A significant red-shift in the absorbance maximum of the OCPA occurs upon deprotonation. The fluorescence emission spectrum of 1 μM CprK using an excitation wavelength of 295 nm is overlayed for a direct comparison.

Previous ligand binding studies have all revealed that CprK binds OCPA with μM affinity. However, individual binding events were observed for *D. dehalogenans* CprK (Pop *et al*., 2006) in contrast to data published for the *D. hafniense* CprK (Joyce *et al*., 2006; Mazon *et al*., 2007). We performed isothermal titration calorimetry (ITC) binding studies that only reveal a single binding event for the *D. hafniense* CprK_C200S_ with a *K*_d_ of 1.4 μm, ΔG of −33.3 kJ mole^−1^ at 298K (Fig. 5A and B). These data are in good agreement with previously observed *K*_d_-values as obtained from Trp fluorescence quenching titration studies (Joyce *et al*., 2006; Mazon *et al*., 2007). It thus appears that a single high affinity binding event can be observed under equilibrium conditions. The marked decrease in fluorescence of the single Trp-106 present in CprK cannot easily be explained by examination of the individual CprK crystal structures that reveal little difference to the immediate Trp-106 environment upon ligand binding (Fig. 2A). However, in contrast to protonated OCPA, the absorption spectrum of deprotonated OCPA overlaps significantly with the Trp-106 fluorescence emission spectrum (Fig. 5C). In view of our previously proposed pK_a_ interrogation model for OCPA binding (Joyce *et al*., 2006), we thus attribute the Trp fluorescence quenching signal to the deprotonation of OCPA following binding and concomitant molecular rearrangement of CprK. We studied the rate of molecular rearrangement by following the rate of Trp-106 fluorescence quenching using stopped-flow methods, and found that a single low affinity binding event can be discerned with a maximum rate of 434 s^−1^ (Fig. 5D). In this case, the 2.6 mM affinity observed for OCPA is within a similar range as that previously indirectly observed for the non-halogenated HPA (6.2 mM, Joyce *et al*., 2006). Our kinetic data therefore indicate weak initial binding to CprK, whereas equilibrium studies show that molecular rearrangement ultimately leads to ∼2000-fold tighter binding of OCPA.

### Extreme positive cooperativity occurs for OCPA binding

**Fig. 6:**
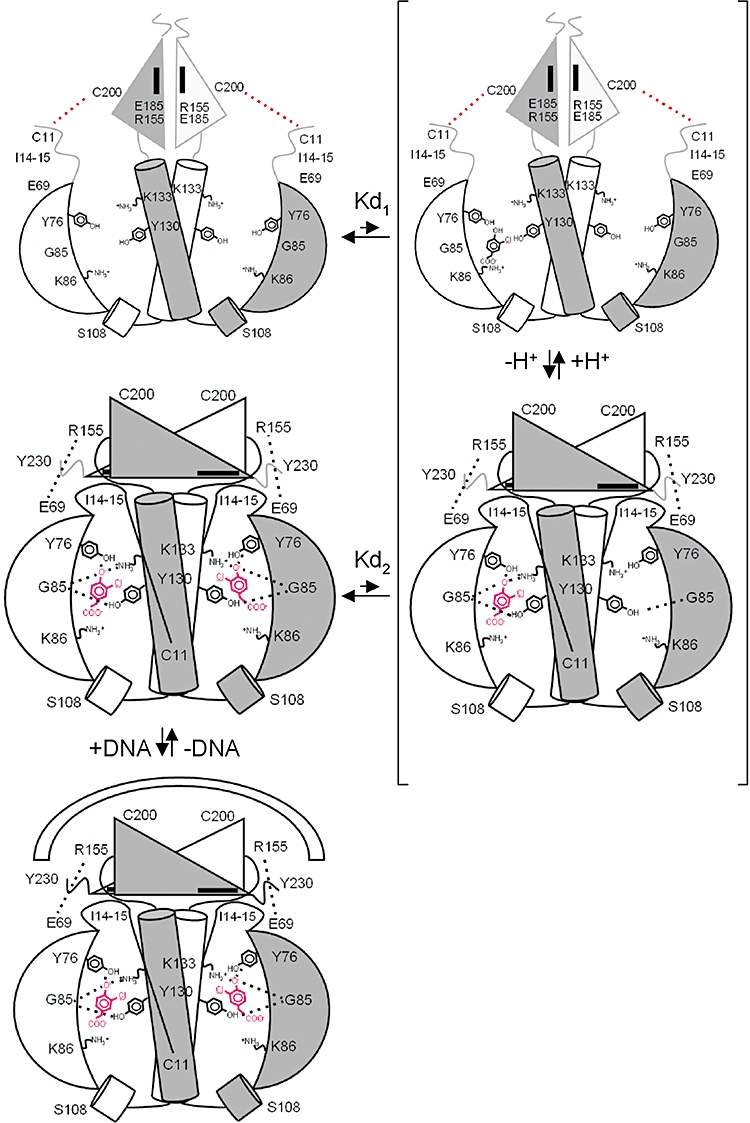
Model for allosteric effects of OCPA binding by CprK. A schematic model illustrating the extreme positive cooperativity model for OCPA binding by CprK and associated structural reorganization. Individual structural elements are depicted in grey scale when observed to be highly mobile. Key amino acids and contacts are indicated where appropriate. For clarity, the DNA-binding domain hydrophobic set of residues involved in domain interactions (Leu-156, Leu-160, Leu-178, Met-176, Ile-186) is depicted as a black rectangle. The bound OCPA molecules are depicted in black (protonated) or red (deprotonated). We postulate binding of the first ligand is characterized by weak binding (*K*d_1_∼2.5 mM) but following ligand deprotonation (as predicted by our pK_a_ interrogation model; Joyce *et al*., 2006) and concomitant reorganization of the entire CprK a high affinity ligand binding site is created for the second ligand (*K*d_2_∼1 μm). It is unclear whether deprotonation occurs prior to or during binding for the second ligand molecule (here depicted as binding in the deprotonated form).

At present, our solution data cannot differentiate between one of two possible models for the CprK:OCPA interaction. The first model assumes no significant cooperativity in binding between both OCPA binding sites occurs, while the second model postulates extreme positive cooperativity for OCPA binding. In the latter case, the relative proportion of CprK:OCPA to either CprK or CprK:OCPA_2_ is negligible under all conditions, which explains why the first weaker binding event cannot be observed under equilibrium conditions. The first model inherently assumes little communication occurs between monomers, which can independently undergo ligand-induced reorganization. This implies a ‘hydrid’ CprK structure with only one monomer in the DNA-binding conformation while the second monomer remains in the ligand-free conformation is possible. However, an overlay of both ligand-bound and ligand-free CprK_C200S_ structures reveals both conformations appear mutually exclusive. In other words, it appears highly unlikely that a CprK dimer could adopt a conformation in which one subunit is in the ligand-bound form while the other remains in the ligand-free conformation. This is due to the fact that the ligand-induced rotation of the sensor domain of one monomer is likely accompanied and/or followed by the reorientation of both the central α-helix and DNA-binding domain of the other monomer. In addition, the associated reorientation of the N-terminal polypeptide stretch, which is needed to stabilize the DNA domain position, can only occur when the β-barrel of the opposite monomer has undergone a rotation. It thus appears from the available structural data that the CprK reorganization upon ligand binding occurs in both monomers as a concerted process, either following binding of the first or the second ligand. Solution data do suggest that binding of the first ligand drives the molecular reorganization of the dimer, implying that a high-affinity binding site is generated in the ligand-free monomer. Recent studies on CRP have revealed that the basis for the observed negative cooperativity for cAMP binding is associated with a pronounced conformational entropic penalty for the second binding event (Popovych *et al*., 2006). In contrast, we assume that in case of CprK the first ligand binding event largely drives molecular rearrangement, with a concomitant decrease in overall flexibility, followed by a second binding event that is not associated with large-scale reorganization (Fig. 6). This distinct behaviour possibly reflects the different physiological roles both transcriptional regulators play. While CRP is known to participate in a large regulatory network, both activating and repressing the expression of many *E. coli* genes (Botsford and Harman, 1992), CprK is postulated to specifically regulate a limited set of operons related to halorespiration. It would thus seem logical that CprK has developed to function as a highly sensitive, single threshold on-off switch while CRP can report on a wide range of cAMP concentrations.

### Crystal structure of the (de)halobox–CprK complex

All of the *Desulfitobacterium* spp. *cpr* operons contain a 5 bp non-perfect inverted repeat with a 4-nucleotide spacer [termed the (de)halobox]. As to the CprK homologues that are specific for the OCPA ligand, Dd-CprK and Dh-CprK, a consensus sequence of (de)haloboxes has been predicted to be TTAAT-N_4_-ATTAA on the basis of sequence alignment (Gabor *et al*., 2006). Indeed, *in vivo* promoter probe experiments using *D. hafiense* CprK paralogues have confirmed binding to this palindromic motif, as well as transcription activation of the downstream gene encoding a beta-galactosidase reporter. Analysis of natural (de)haloboxes that deviated from the consensus revealed significantly decreased rates of reporter expression (Gabor *et al*., 2006).

The 1.8 Å crystal structure of CprK_C200S_ in complex with a palindromic 30 bp dsDNA fragment containing the consensus (de)halobox sequence (as determined by *in vivo* promoter analysis; DNA sequence CCGGCATG**TTAAT**GCGC**ATTAA**CATGCCGG, (de)halobox consensus sequence in bold) could be obtained by cocrystallization (Fig. 1). The asymmetric unit contains a single CprK monomer bound to the corresponding half of the (de)halobox DNA, with the CprK dimer and complete (de)halobox DNA related by crystal symmetry. No significant changes could be observed for the majority of the CprK structure when comparing the DNA-bound form with the CprK_C200S_:OCPA complex structures. However, in contrast to the latter, the CprK C-terminus region has become structured and is now in close contact with the DNA (de)halobox. The majority of the dsDNA fragment is clearly visible with exception of the outermost nucleotides (1–3 and 28–30) that do not contribute to the CprK–DNA interface.

**Fig. 7:**
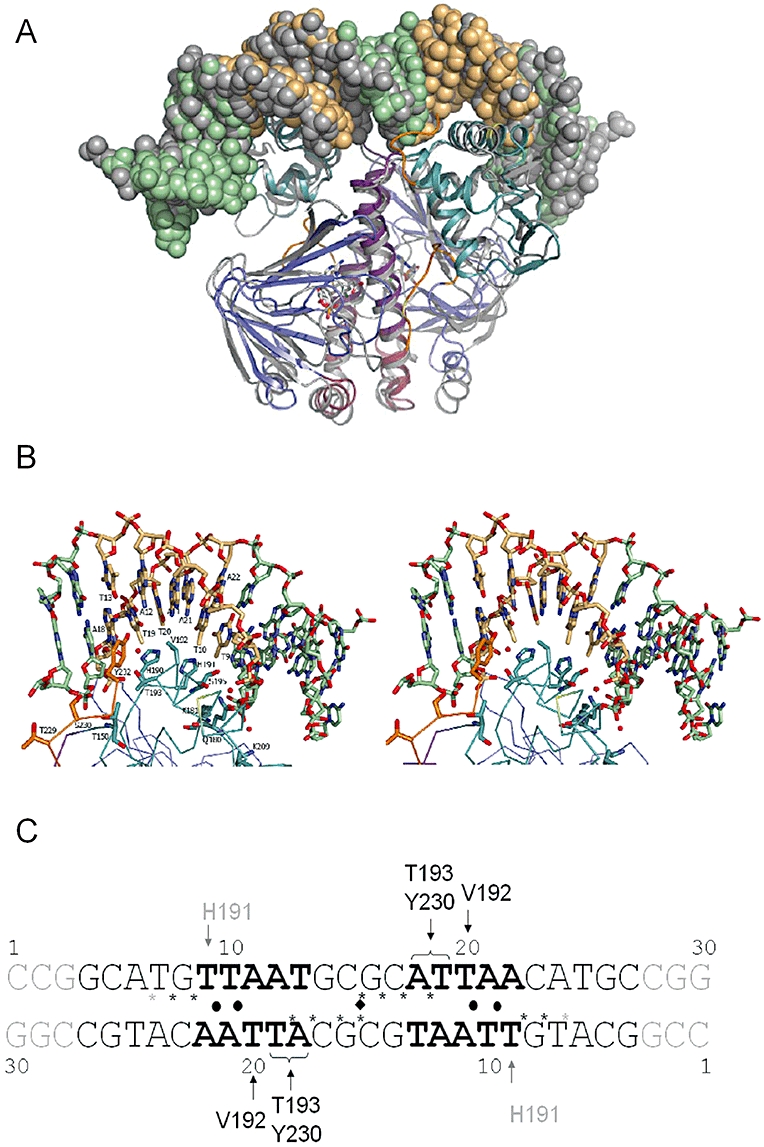
(de)halobox DNA binding by CprK_C200S_:OCPA. A. An overlay of the CprK_C200S_:OCPA:DNA crystal structure with the available CRP:cAMP:DNA structure (PDB code 2CGP). The overlay is based on structural alignment of the respective HTH motives. CprK and associated DNA are depicted and colour coded as in Fig. 1. The CRP is similarly represented but in grey tones. B. Stereoview of DNA–CprK interface contact area. The DNA and key amino acids are represented in atom coloured sticks with carbons coloured according to Fig. 1. C. Schematic overview of the various CprK_C200S_ DNA contacts established. The (de)halobox consensus sequence is depicted in bold while nucleotides that were not observed in the structure are depicted in grey scale. A black sphere indicates the base pairs for which geometric parameters deviate significantly from average. DNA backbone phosphates that make direct contact with CprK residues are depicted by a star.

Overall, the complex is highly similar to the CRP–DNA complexes available (Lawson *et al*., 2004; Fig. 7A). As observed with CRP–DNA complexes, the DNA helix displays significant curvature (to approximately 80°) with the individual hinge points occurring at consensus sequence positions. A large proportion of the polar contacts established at the protein–DNA interface are made between the phosphate backbone and the protein, a subset that includes contacts made with residues from the C-terminal region (residues 227–232) that is disordered in the DNA-free structures. However, in contrast to CRP, few polar contacts are made between CprK and the individual DNA bases (Fig. 7B and C). The C-terminal Tyr-230 together with Thr-193 establishes a hydrogen bond network with A18 and T19 that includes a structural water molecule. It is likely that this particular interaction is responsible for the requirement of a purine and a pyrimidine at positions 18 and 19 respectively. The homologous CRP lacks this interaction as it does not possess a corresponding C-terminal region, explaining why the CRP promoter region contains a 6-nucleotide spacer between the palindromic motifs; in contrast, the CprK consensus sequence contains a 4-nucleotide spacer, as does the FNR-box with FNR containing a similarly extended C-terminus. In addition, a putative water-mediated contact between His-191 (HTH recognition helix) and T9 is possible, although not directly observed. Apart from these interactions, no other nucleotide-specific polar contacts could be observed. In contrast, the hydrophobic contacts established between Val-192 of the recognition helix and T20 clearly leads to the strict requirement for a pyrimidine at this position (Fig. 7B). Previous mutagenesis work on CprK and FNR revealed that a Val at position 192 (Glu-209 in FNR) is indeed linked to specificity for a pyrimidine at position 20. All (de)halobox sequences that are presently available contain a T at position 20, strongly contributing to specificity and avoiding cross-talk with FNR-like regulators. In fact, the latter recognize a consensus sequence that differs from the consensus (de)halobox only by virtue of a C at the corresponding position (Gabor *et al*., 2006).

No significant direct contact is made between CprK and the A21:T10 A22:T9 base pairs that are strictly conserved in (de)haloboxes. However, it is possible that the above-mentioned His-191, which is conserved in Dd-CprK and Dh-CprK1, contributes to the specificity to some extent. More importantly, the conformations of both the T20:A11 A21:T10 and A21:T10 A22:T9 base pair steps are significantly perturbed by protein binding when compared with average values for both B-DNA and protein–DNA complexes (Olson *et al*., 1998). The T20:A11 A21:T10 base pair step roll parameter (for a definition of base pair geometric parameters see Olson *et al*., 2001) is significantly higher than those observed on average (16 as opposed to 1.1 ± 4.9) while the twist and rise parameters for the A21:T10 A22:T9 base pair step are 26° and 2.8 Å, respectively, as opposed to values of 35.1 ± 3.9° and 3.27 ± 0.22 Å that are more usually observed. The energetic costs associated with the specific deformations here observed are predicted to be minimal for the case of A:T A:T base pairs as opposed to any other set of base pairs (Olson *et al*., 1998). This provides a likely explanation for the strict requirements for T at positions 9 and 10 and corresponding A at positions 21 and 22, as other bases at these positions would lead to a prohibitively large energy barrier towards the DNA deformation that is required for tight binding to CprK. Similar to CRP, the CprK sequence specificity is in large part due to an indirect readout mechanism (Lawson *et al*., 2004).

## Conclusions

The availability of crystal structures for all individual CprK states in addition to both kinetic and thermodynamic data for ligand binding allows the construction of a detailed model for the activation mechanism of the CprK transcriptional regulator that controls the expression of halorespiration genes (Fig. 6). In addition, CprK is the first member of the widespread CRP-FNR family for which a complete atomic description of all individual allosteric states is now available (Fig. 1). We show that, in absence of ligand, CprK is a flexible molecule with both DNA-binding domains essentially organized into a single unit by virtue of predominantly hydrophobic interactions (Fig. 3A). This unit appears largely unrestrained in motion, as are the individual N-terminal polypeptide segments (residues 1–18). However, the Cys-11 and Cys-200 residues of opposite monomers, respectively, part of each of these mobile structural elements, are likely to be in close proximity for most of the time. This explains the marked susceptibility of CprK for inactivation by oxygen through formation of the intermolecular Cys-11–Cys-200 disulphide bridges. While it remains unclear whether this particular property has any physiological relevance, the mechanism by which it here occurs can serve as a model for other systems that are regulated by similar oxidation/reduction events. In case of CprK, oxidation leads to severe constraints on the position of the DNA-binding domains and hence a reduction in overall flexibility (Fig. 4A). Furthermore, while it does appear to not significantly affect ligand binding mode (13–14 and this work), it prohibits formation of the appropriate inter-domain contact network that establishes the DNA-binding form, thus inactivating transcription under aerobic conditions.

In contrast, ligand binding to the reduced CprK leads to a concerted reorganization of both monomers that moves the DNA-binding domains in positions compatible with binding to the palindromic (de)halobox sequence (Fig. 6). This reorganization follows an initial induced-fit binding of OCPA that ultimately affects the position of two key sets of residues: Glu-68–Asp-69 from the sensor domain β-barrel and the N-terminal residues Ile-14–Ile-15–Pro-16. The first set of amino acids is displaced from the original position occupied via a rigid body hinge motion of the sensor domain β-barrel. This motion is driven by docking of the chloride atom in the binding pocket provided by the central α-helices and formation of a tight phenolate–Lys-133 interaction, possibly concomitant with ligand deprotonation. A further consequence of ligand binding is the ordering of the N-terminus that positions residues Ile-14–Ile-15–Pro-16 to stabilize the DNA-binding conformation through interactions with the DNA-binding domains (Fig. 3B and C). In this case, the structure of OCPA-soaked CprK_C200S_ suggests that the N-terminus reorganization does not occur as a direct consequence of ligand binding, but after or concomitant with DNA domain reorientation. The N-terminal region has been postulated to be essential to the mechanism of the related CO sensor CooA (Borjigin *et al*., 2007). In this case, a recent crystal structure of the CO-bound form of a *Carboxythermus hydrogenoformans* CooA mutant revealed how CO binding displaces the N-terminal Pro residue from the haem co-ordination sphere and thus allows the N-terminus to reorient and serve as a bridge between the sensor and DNA-binding domains. Unfortunately, in this case the physiological relevance of that crystal structure is a matter for debate given the lack of a haem cofactor in one of the CooA monomers.

Concomitant with DNA binding to CprK, the C-terminus becomes ordered and establishes nucleotide-specific contacts that lead to the observed specificity for A and T at positions 18 and 19 (Fig. 7B). Only a single residue of the HTH motif recognition helix, Val-192 confers an additional sequence requirement for T/C on position 20, which is fulfilled by T features in all of the currently known (de)halobox sequences. The strict conservation of the T9-T10/A21-A22 appears predominantly a consequence of indirect readout, given that this particular region of the DNA is highly deformed upon CprK binding.

CprK has a marked specificity for the halogenated ligand compared with the non-halogenated product of halorespiration, both in terms of affinity and functionality. Our data confirm that this is not merely a consequence of the physical presence of the additional bulk of the halogen atom, but the specific requirement of binding to the deprotonated phenolic ligand concomitantly with or prior to molecular rearrangement. Halogenation of the phenol group at ortho- and para-positions indeed leads to a significant increase in the acidity of the phenolic group compared with the non-halogenated version. Furthermore, we postulate that binding occurs with extreme positive cooperativity, such that relatively weak binding of the first ligand leads to extremely tight binding of the second ligand and sequence-specific DNA-binding (Fig. 6).

It will come as no surprise that the key amino acids underpinning the CprK allosteric mechanism are distinct from those indicated to be important for other structurally characterized members of the CRP-FNR family (Lanzilotta *et al*., 2000; Passner *et al*., 2000; Harman, 2001; Green *et al*., 2001; Benoff *et al*., 2002; Korner *et al*., 2003; Lawson *et al*., 2004; Eiting *et al*., 2005; Borjigin *et al*., 2007), given the difference in ligand nature as well as distant evolutionary relationship. Given that the actual position of the ligand binding site is well conserved in CprK and CRP, the crucial difference most likely is the different positions of – and network of interactions established between – the sensor and HTH domains within the dimers of CprK and CRP; these different architectures require different mechanisms to rearrange. Nevertheless, comparison between individual crystal structures and/or modelling of CRP-FNR members in distinct states (e.g. CRP and CooA) has been used to guide further research and provide mechanistic understanding. However, the fact that CprK differs significantly from the well-studied CRP in terms of ligand binding characteristics and inter-domain contacts clearly warrants against this. Within the CRP-FNR superfamily, functionally distinct transcriptional regulators have thus evolved based on a common modular design: ligand binding β-barrels attached to a central α-helix Leu-zipper pair that in turn is connected to HTH containing domains. The exact mechanism by which ligand binding within the β-barrels leads to DNA-binding, however, depends on the exact nature of the ligand induced β-barrels domain reorganization and on the allosteric networks that establish communication between ligand- and DNA-binding domains. The versatility of this design has enabled the control of a wide range of processes by members of this superfamily, including oxidative stress response, stationary phase survival, nitrogen fixation, denitrification, pathogenesis and quorum sensing. This study has elucidated the mechanism by which halorespiration is controlled by CprK and contributes significantly to understanding the mechanism of allosteric regulation of transcriptional regulators. Nevertheless, further study into transcriptional regulation of halorespiration is required as a majority of reductive dehalogenases is associated with two-component or MarR-like regulators (Kube *et al*., 2005; Seshadri and *et al.*, 2005), while halorespiration also occurs with non-phenolic halogenated substrates.

## Experimental procedures

### Protein purification

The *D. hafniense* CprK and CprK_C200S_ were produced and purified as described previously (Gabor *et al*., 2006; Joyce *et al*., 2006).

### Tryptophan fluorescence quenching

Rapid rate binding kinetics were measured by using a SX1185 stopped-flow spectrophotometer (Applied Photophysics). The quenching of the tryptophan fluorescence was followed by using an excitation wavelength of 295 nm and a 320 nm cut-off filter on the emission detector. C200S CprK (final reaction cell concentration of 1 μm) in 50 mM potassium phosphate buffer (pH 7) was mixed with a range of OCPA concentrations at 25°C. For each ligand concentration, between 6 and 10 replicate measurements were collected and averaged. Transients were analysed using Spectrakinetics software (Applied Photophysics) and were fitted to a single exponential equation. The kinetic parameters were obtained by fitting the rate constants to the following equation: *k*_obs_ = *k*_max_[S]/(*K*_m_ + [S]), where *k*_obs_ is the observed rate constant at each OCPA concentration, [S] is the concentration of OCPA and *k*_max_ is the maximum rate constant as [S] approaches ∞.

### Isothermal titration calorimetry

All the titration experiments were performed using the VP-ITC system (MicroCal, Northampton, MA). In each experiment 49 aliquots of 6 μl containing 0.24 mM OCPA were injected into 1.4348 ml of CprK (0.01 μm in 50 mM potassium phosphate buffer pH 7.5, 150 mM sodium chloride, 10% glycerol) at a temperature of 25°C. Heats of dilution were determined by titration of OCPA into buffer. These heats were deducted from the experimental data prior to the data being fitted. Titration data were fitted using a one site non-linear least squares curve fitting algorithm with three floating variables: stoichiometry (*n*), binding constant (*K*_b_) and change in enthalpy of interaction (ΔH). OCPA binds to CprK with a stoichiometry of 0.95.

### Crystallization of ligand-free CprK_C200S_

Small clusters of ligand-free CprK_C200S_ crystals were obtained using the hanging drop vapour diffusion method using a reservoir solution of 100 mM MES pH 6, 10% MPD with drops made from 2 μl 10 mg ml^−1^ CprK_C200S_ plus 2 μl reservoir solution, incubated at 294K. A microseeding protocol using similar conditions led to significantly larger and unique crystals. Prior to flash-cooling, crystals were briefly immersed in 20% MPD, 100 mM MES pH 6. When soaking ligand-free CprK_C200S_ crystals with halogenated phenol acetic acid compounds, the crystals were soaked for approximately 10 min in mother liquor supplemented with 100 mM of the corresponding compound.

### Crystallization of the CprK_C200S_:OCPA complex

Crystals of the CprK_C200S_:OCPA complex were routinely obtained by placing a 4 μl drop of 10 mg ml^−1^ CprK_C200S_ in presence of 1 mM OCPA above a reservoir solution that contained 200 mM NaCl, 100 mM sodium acetate pH 5, 20% PEG 6000 and incubating at 277 K. Prior to cryocooling crystals were briefly immersed in a drop containing 10 mg ml^−1^ CprK_C200S_ in presence of 1 mM OCPA and either 15% glycerol or 10% PEG 200 that had been equilibrated against the same reservoir solution.

### Crystallization of ligand-free, oxidized CprK

Crystals for the oxidized, ligand-free WT CprK were obtained using the hanging drop vapour diffusion method using 100 mM CaCl_2_, 100 mM HEPES pH 7, 10% PEG 2000 MME as the reservoir solution. Drops were made by mixing 2 μl of 10 mg ml^−1^ WT CprK with 2 μl of reservoir solution and incubating at 293K.

### Crystallization of the CprK_C200S_ in complex with DNA and OCPA

A perfect palindromic 30 bp oligonucleotide with sequence CCGGCATGTTAATGCGCATTAACATGCCGG was prepared prior to crystallization by heating to 367K before slow cooling to room temperature. Crystallization was achieved using a DNA : CprK_C200S_ molar ration of 1.2:1 in presence of 1 mM OCPA. Crystal were obtained using the sitting drop vapour diffusion method at 277 K using a well solution of 0.17 M ammonium acetate, 0.085 M sodium acetate pH 4.6, 25.5% PEG 4K and 15% glycerol.

### Data collection and structure elucidation of CprK crystal structures

Data for all crystal structures were collected to respective resolutions from single cryofrozen crystals at ESRF Grenoble beamlines. The data were scaled and integrated using the XDS package (Kabsch, 1988) and subsequently handled using the CCP4 suite (Collaborative Computational Project, Number 4, 1994). All structures were solved by either molecular replacement using Phaser or difference fourier methods. Individual effector and DNA domains were used as search models for molecular replacement. Refinement and model building were carried out using Refmac 5 (Murshudov *et al*., 1997) and COOT (Emsley and Cowtan, 2004). Data and final refinement statistics are given in Table 1. Coordinates and structures factors have been submitted to the PDB database under codes 3E5U, 3E5X, 3E5Q, 3E6B, 3E6C, 3E6D.

**Table 1:** Crystallographic data collection and refinement statistics.

	OCPA-1	OCPA-2	Unbound	OCPA-soaked	Unbound-oxidised	DNA complex
Space group	P2_1_	P2_1_	P2_1_2_1_2_1_	P2_1_2_1_2_1_	P2_1_	P6_2_22
Unit cell (Å)	*a* = 56.1	*a* = 56.7	*a* = 42.9	*a* = 42.5	*a* = 79.4	*a* = 100.6
	*b* = 117.2	*b* = 118.4	*b* = 86.8	*b* = 86.5	*b* = 64.7	*c* = 149.7
	*c* = 84.9	*c* = 87.5	*c* = 124.4	*c* = 125.2	*c* = 148.4	
	*β* = 94.7	*β* = 95.7			*β* = 105.3	
Resolution (Å)	1.8	2.0	2.0	2.0	3.2	1.8
Total reflections	226 615	241 727	106 603	128 807	43 490	249 774
Unique reflections	99 029	75 930	28 123	29 866	19 900	35 026
Completeness percentage	97.8 (99.4)	98.0 (97.5)	81.5 (85.8)	94.8 (84.1)	82.0 (84.3)	96.7 (96.8)
Redundancy	2.3 (2.2)	3.2 (3.2)	3.8 (3.8)	4.3 (3.6)	2.2 (2.2)	7.1 (7.3)
*R*_merge_	7.4 (37.7)	5.4 (27.1)	12.8 (50.8)	10.0 (54.6)	16.5 (57.4)	11.1 (35.4)
I/sigI	7.5 (2.0)	13.7 (3.6)	9.6 (2.4)	11.3 (2.7)	7.3 (1.9)	13.5 (3.7)
*R*_work_	18.2	19.3	21.3	18.9	25.8	21.7
*R*_free_	22.7	23.8	25.9	23.1	31.7	25.0
RMSD angle (°)	1.87	1.92	1.73	1.89	2.39	2.37
RMSD length (Å)	0.021	0.023	0.021	0.025	0.024	0.021
Overall B (Å_2_)	28.7	32.3	35.8	31.2	80.2	58.3
Water B (Å_2_)	37.9	44.8	35.9	40.7	NA	49.6
OCPA B (Å_2_)	21.6	48.9	NA	23.2	NA	23.0
PO4 B (Å_2_)	47.2	NA	NA	NA	NA	NA
DNA B (Å_2_)	NA	NA	NA	NA	NA	84.7

NA, not applicable.
